# Deregulation of Microcephalin and ASPM Expression Are Correlated with Epithelial Ovarian Cancer Progression

**DOI:** 10.1371/journal.pone.0097059

**Published:** 2014-05-15

**Authors:** Rawiah Alsiary, Anke Brüning-Richardson, Jacquelyn Bond, Ewan E. Morrison, Nafisa Wilkinson, Sandra M. Bell

**Affiliations:** 1 Leeds Institute of Biomedical and Clinical Sciences, University of Leeds, Leeds, United Kingdom; 2 Leeds Institute of Cancer and Pathology, University of Leeds, Leeds, United Kingdom; 3 St James's Institute of Oncology, Leeds Teaching Hospitals NHS Trust, Leeds, United Kingdom; MD Anderson Cancer Center, United States of America

## Abstract

Mutations in the *MCPH1* (Microcephalin) and *ASPM* (abnormal spindle-like microcephaly associated) genes cause primary microcephaly. Both are centrosomal associated proteins involved in mitosis. Microcephalin plays an important role in DNA damage response and ASPM is required for correct division of proliferative neuro-epithelial cells of the developing brain. Reduced *MCPH1* mRNA expression and *ASPM* mRNA over-expression have been implicated in the development of human carcinomas. Epithelial ovarian cancer (EOC) is characterised by highly aneuploid tumours. Previously we have reported low Microcephalin and high ASPM protein levels and associations with clinico-pathological parameters in malignant cells from ascitic fluids. To confirm these previous findings on a larger scale Microcephalin and ASPM expression levels and localisations were evaluated by immunohistochemistry in two cohorts; a training set of 25 samples and a validation set of 322 EOC tissue samples. Results were correlated to the associated histopathological data. In normal ovarian tissues the Microcephalin nuclear staining pattern was consistently strong. In the cancer tissues, we identified low nuclear Microcephalin expression in high grade and advanced stage tumours (*p*<0.0001 and p = 0.0438 respectively). ASPM had moderate to high nuclear and low to moderate cytoplasmic expression in normal tissue. Cytoplasmic ASPM expression decreased with tumour grade and stage in the serous subtype of EOC (p = 0.023 and p = 0.011 respectively). Cytoplasmic ASPM increased with tumour stage in the endometrioid subtype (p = 0.023). Increasing tumour invasiveness (T3) and lymph node involvement (N1) also correlated with a decrease in cytoplasmic ASPM in EOC (p = 0.02 and p = 0.04 respectively). We have validated previous findings of deregulated expression of Microcephalin and ASPM in EOC by confirming associations for low nuclear Microcephalin levels and high cytoplasmic ASPM levels in a larger scale tumour tissue study. Microcephalin and ASPM may prove useful biomarkers in EOC.

## Introduction

Approximately 225,000 new cases of ovarian cancer were diagnosed in 2008 worldwide, comprising 4% of all female cancers [1], [2]. The majority of ovarian cancers are epithelial, frequently present at advanced stages and are associated with high mortality rates [3]. Therefore, investigating the function and expression of potential prognostic proteins is essential for the advancement of our understanding of the molecular pathogenesis of epithelial ovarian cancer (EOC). This is especially desirable with regards to the identification of diagnostic biomarkers for patient management [4].

The *MCPH1* and *MCPH5* genes encode Microcephalin and the abnormal spindle-like microcephaly-associated protein (ASPM) respectively [5], [6]. *MCPH1* and *MCPH5* are two of ten microcephaly genes identified, which are implicated in autosomal recessive primary microcephaly (MCPH) [7]–[13]. Microcephaly is characterized by reduced foetal brain growth resulting from mitotic defects during embryonic brain development [14]. Microcephalin is a nuclear and cytoplasmic protein consisting of 835 amino acids. The protein contains three BRCA1 C-terminus domains (BRCT), one *N*-terminally located and two in the *C*-terminus [5]. Microcephalin is involved in DNA damage response with a further role as a regulator of chromosome condensation preventing cells from entering mitosis before DNA replication is completed [15]–[17]. Microcephalin is also known as BRIT1 (*BRCT*-repeat inhibitor of hTERT expression), which was initially identified as a transcriptional repressor of human telomerase reverse transcriptase (hTERT), the catalytic subunit of human telomerase [18]. The ASPM protein consists of 3477 amino acids [6] organised into a putative *N*-terminal microtubule binding domain [19], [20], two Calponin homology repeat motifs, over 80 isoleucine-glutamine (IQ) repeats [6], [21] which typically bind calmodulin and a *C*-terminus consisting of a single Armadillo like sequence and a region involved in cytokinesis [8], [22]. ASPM plays a role in controlling mitotic spindle function [6], [22]. ASPM is located at the spindle poles in metaphase and at the nucleus and centrosome during interphase [6], [22]–[27]. Furthermore, ASPM localizes to the midbody during cytokinesis, suggesting that it plays a role in abscission [22], [27].

Several lines of evidence indicate that Microcephalin and ASPM play a role in carcinogenesis. At the DNA level, Rai *et al.* reported that *MCPH1* copy number was decreased in 40% (35/87) of advanced EOC and in 72% (39/54) of breast cancer cases [16]. Similarly, at the mRNA level *MCPH1* mRNA was decreased in 63% (19/30) of EOCs [16]. We have reported reduced Microcephalin protein levels in 29% (93/319) of invasive ductal breast carcinomas, with Microcephalin expression decreasing with increasing breast cancer grade. Importantly, Microcephalin was an independent predictor of overall breast cancer specific survival [28]. Recently two further small breast cancer studies have confirmed the association of reduced Microcephalin expression with tumour progression and prognosis [29], [30]. Decreased Microcephalin expression was also reported in a small prostate cancer study [16], which suggested that a negative correlation exists between Microcephalin levels, genomic stability and chromosomal aberration. Recently inactivation of *MCPH1* by deletion, promoter methylation and mutation was identified in an oral squamous cell cancer study [31]. This study also showed that Microcephalin over expression inhibited proliferation, invasion and anchorage independent growth and tumour growth in nude mice supporting the tumour suppressor function of Microcephalin [31].

In contrast, *ASPM* mRNA levels were increased in tumour and transformed human cells [26], [32]. Increased *ASPM* mRNA and protein levels were also identified in glioblastoma multiforme (GBM), where they were associated with increasing tumour grade [32], [33]. In addition, *ASPM* mRNA upregulation was identified in 66% (162/247) of hepatocellular carcinomas, an observation associated with increased invasion, high stage and early tumour recurrence [34]. Recently upregulation of ASPM correlated with reduced patient survival has also been identified in pancreatic cancer [35].

Our recent EOC study determined a correlation between Microcephalin and ASPM levels with tumour grade and survival in cell lines and in primary cultures of malignant cells derived from ovarian ascites samples [36]. In this work we have validated our original findings in a larger scale study utilising EOC tissue samples and have investigated the roles of Microcephalin and ASPM in EOC progression. Our results suggest that Microcephalin and ASPM may be useful biomarkers in EOC management.

## Materials and Methods

### Ethics Statement

Appropriate ethical approval was obtained from the Local Research Ethics Committee of the Leeds Teaching Hospitals NHS Trust, Leeds, UK, (REC reference 09/H1306/96). All participants provided written informed consent and all data were analysed anonymously.

### Patient Samples

A cohort of 25 tumour archival, formalin-fixed, paraffin-embedded (FFPE) blocks were obtained from the histopathology department of St James's University Hospital, Leeds and used to create the training set. The sample set in this cohort represented four major (most frequently encountered) EOC subtypes (serous, endometrioid, mucinous and carcinosarcoma) and were predominately grade 3 tumours. All the blocks in this cohort were then combined into one in-house tissue microarray (TMA). A normal cohort consisted of 16 ovarian tissue samples taken from either normal ovary or from normal tissue adjacent to tumour plus 4 normal fallopian tube and 4 normal endometrium samples were also available.

Another independent cohort of 322 EOC samples was obtained from Tissue Array Networks and was designated the validation set. The validation set consisted of 294 different samples of five major EOC subtypes (serous, endometrioid, adenocarcinoma, mucinous and clear cell), plus 8 normal ovarian tissues taken from normal ovary and 20 from normal tissue adjacent to tumour. The 322 cases were represented in 616 cores, with double cores for cancer cases and a single core for normal tissue. Tissue cores were 0.6 mm in diameter with a thickness of 5 µm. Clinical data (grade, pathology diagnosis, International Federation of Gynaecology and Obstetrics (FIGO) staging and TNM staging [37]) were available for this cohort. The mean age of the 294 patients was 48 years (range: 19 to 76 years). The results for association with age were obtained after the data were dichotomized into two groups (>50 and ≤50). Detailed patient characteristics of the validation set are summarized in Table 1.

**Table 1:** Patients' characteristics in the validation set.

Malignant cases in the validation set *n* = 294 (%)	
**Age**	
Range	19–76
Mean	48
**Age distribution**	
<50	125 (42.5)
≥50	169 (57.5)
**Tumour grade**	
G1	63 (25.7)
G2	72 (29.4)
G3	110 (44.9)
G2/G3	7
Data Missing	42
**Histological tumour type**	
Serous adenocarcinoma	209 (71.1)
Mucinous adenocarcinoma	45 (15.3)
Endometrioid adenocarcinoma	12 (4)
Adenocarcinoma	15 (5.1)
Clear cell carcinoma	13 (4.4)
**Tumour FIGO stage**	
I	205 (69.7)
II	42 (14.3)
III	35 (11.9)
IV	12 (4.1)
**Tumour TNM stage**	
**Primary tumour invasion (T)**	
T1	207 (70.4)
T2	59 (20.1)
T3	28 (9.5)
**Lymph node status**	
No regional lymph node metastasis (N0)	256 (87)
Metastasis in 1–3 region lymph node (N1)	36 (12.3)
Regional lymph node cannot be assessed (NX)	2 (0.7)
**Distant metastases**	
No (M0)	282 (95.9)
Yes (M1)	12 (4.1)

### Tissue Microarray Construction

An in-house TMA was constructed from 25 EOC samples at the Leeds Institute of Cancer and Pathology facility following a protocol described by Ellis *et al* [38]. Manual tissue arrayer punches with a diameter of 0.6 mm (Beecher Instruments, Inc) were used to create the cores. Tissue cores were selected from the most representative tumour area in each tumour block after review of the associated haematoxylin and eosin stained slides by the specialist gynaecological histopathologist (NW).

### Immunohistochemistry Detection of Microcephalin and ASPM

To optimise the Microcephalin and ASPM staining protocol, the in house EOC TMA sections were stained using a range of antibody dilutions and antigen retrieval methods. Microcephalin and ASPM were stained separately under the same conditions except for the antibody retrieval and dilution. Slides were deparaffinised and rehydrated using xylene and ethanol series. To block hydrogen peroxidase activity, slides were soaked in 3% H_2_O_2_ in methanol. Antigen retrieval was carried out using Vector antigen retrieval buffer (Vector Laboratories Ltd) in a pressure cooker for 4 minutes for Microcephalin and 2 minutes for ASPM. Sections were blocked with 1∶10 vector casein solution (Vector Laboratories) to reduce non-specific staining. On the TMA sections the rabbit anti-Microcephalin antibody ab2612 (Abcam) was used at 1/300 and the rabbit anti-*N*-terminal ASPM antibody 216.1 [36] used at 1/400. The slides were incubated at 4°C overnight in a humidified chamber. After three washes with TBS Tween (0.1%) the slides were incubated for 30 minutes at room temperature with the rabbit/mouse secondary, peroxidase-conjugated EnVision polymer (DAKO). After further washes the reaction was visualized by the addition of 2% diaminobenzine+ Chromogen in DAP substrate buffer for 10 minutes (DAKO). Sections were counterstained with Mayer's haematoxylin (VWR International Ltd). Negative controls, without primary antibody and positive controls of normal ovarian tissue were included in each batch of immunohistochemistry.

### Microcephalin and ASPM Antibody Validation

In order to determine the staining specificity of the Microcephalin antibody on FFPE samples, an immunizing peptide blocking experiment was performed on EOC tissue sections and on the 25 core in-house TMA. The antibody was neutralized by pre-absorption with the BRIT1 peptide ab13737 (Abcam). The antibody was diluted to its predetermined working concentration (described above) and incubated with a tenfold excess of peptide overnight at 4°C before application to the tissue. Tissue sections with neutralized antibody were compared with corresponding tissue sections stained with antibody alone.

We had already determined the antibody specificity of the ASPM antibody 216.1 in previous work by peptide blocking immunofluorescence experiments [36]. We further confirmed the staining pattern of the antibody observed in IHC by staining the same in-house EOC TMA with another antibody (279.3) generated against the *C*-terminus of ASPM. Similar staining results were obtained with both antibodies with only slight differences between cytoplasmic staining levels.

For further validation of the specificity and sensitivity of the antibodies and to control for the effects of formalin fixation and antigen retrieval, *MCPH1* and *ASPM* were knocked down in the U-2 OS cell line (ATCC, Manassas, VA, USA) by siRNA as previously described [17], [36], [22]. After 72 hrs cells were harvested, pelleted, formalin fixed and suspended in 1% agar then paraffin embedded and used as controls for antibody validation.

### Immunohistochemical Evaluation

All the TMA sections were scanned using the Aperio Scan Scope slide scanner (Aperio Technologies) at 40x magnification, using morphometry tools in Imagescope software (Aperio). For both Microcephalin and ASPM the replicate cores for each sample were scored for staining intensities of nuclear and cytoplasmic staining (intensity score, 0 =  no staining, 1 =  weak staining, 2 =  moderate staining and 3 =  strong staining) as well as the proportion of cells with positive nuclear or cytoplasmic staining within the cores (proportion score). To analyse the Microcephalin staining the semi-quantitative Allred (quick) 8-unit system was used [39]. The proportion of positive nuclear or cytoplasmic stained cells within each core was defined as 0 =  no staining, 1 = <1% staining, 2 = 1–10% staining, 3 = 11–33% staining, 4 = 34–66% staining, 5 = 67–100% staining. The final score was obtained by adding the intensity scores to the proportion scores, achieving a minimum score of 0 and a maximum score of 8. Cases with a score of 4 or lower were called low, while cases with a score of 5 or 6 were labelled as moderate and samples with a score of 7 or 8 were considered as expressing high levels of Microcephalin. For data analysis of ASPM expression a slightly more sensitive data analysis system was used, in which the staining intensity was multiplied by the percentage of cells with nuclear or cytoplasmic staining [40]. Thus the lowest score was 0 and the highest 300. Samples with a score of 0 were called negative, samples with a score of 1–100 low, samples with a score of 101–200 moderate and samples with a score of 201–300 were scored as high expression levels. For discontinuous data analysis all negative and low samples were designated low and all medium and high samples were designated high ASPM levels.

### Statistical Analysis

The non-parametric Kruskall-Wallis (K-W) and Wilcoxon-Mann-Whitney (W-M-W) test were used to identify the association between Microcephalin and ASPM with clinical variables (disease stage, grade, histology type, metastases, lymph node involvement and age of patients). All statistical tests were two-sided, and a *P*≤0.05 values was considered statistically significant.

## Results

### Microcephalin and ASPM Antibody Characterization

**Figure 1:**
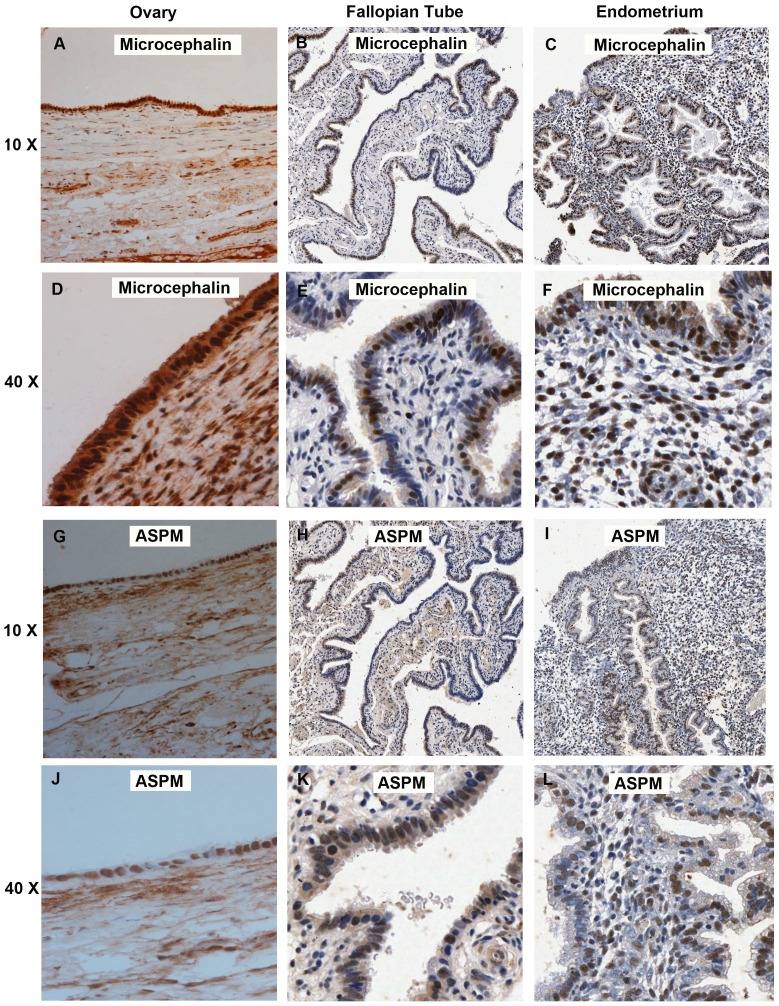
Immunohistochemical analysis of Microcephalin and ASPM in normal tissue samples. Normal ovarian epithelium demonstrating strong Microcephalin expression in both the nuclei and cytoplasm (A & D) and strong nuclear and moderate cytoplasmic ASPM expression (G & J). Normal Fallopian tube demonstrating heterogeneous moderate nuclear and weak cytoplasmic Microcephalin staining (B & E) and ASPM staining demonstrating strong nuclear and moderate cytoplasmic expression (H & K). Normal Endometrium showing heterogeneous strong nuclear and cytoplasmic Microcephalin staining (C & F) and strong nuclear and weak cytoplasmic ASPM staining (I & L). All images are x15 magnification.

**Figure 2:**
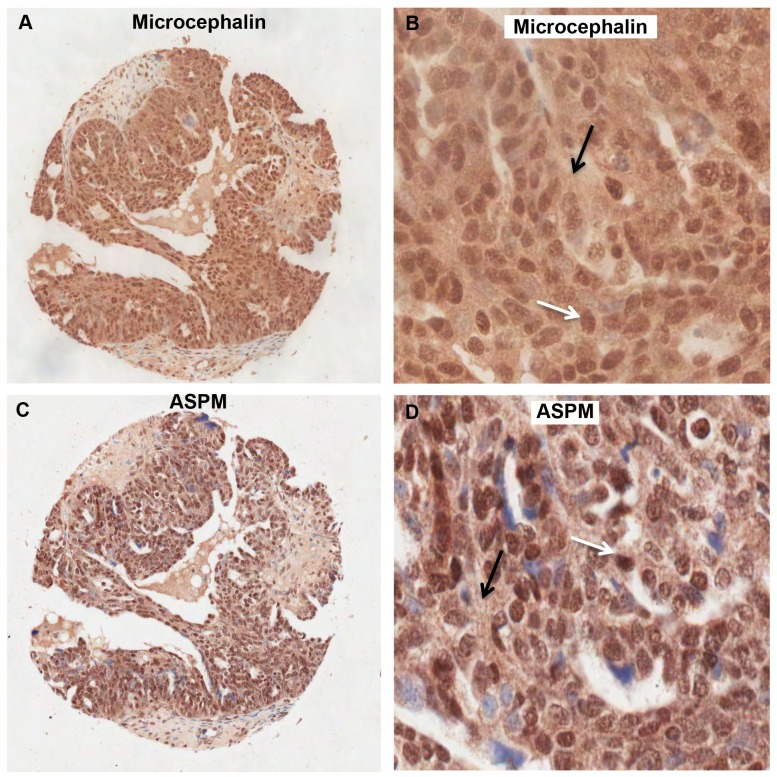
Immunohistochemical analysis of Microcephalin and ASPM expression in malignant samples. Serous adenocarcinoma TMA cores showing nuclear and cytoplasmic Microcephalin (A and B) and ASPM expression (C and D) respectively. White arrow shows nuclear staining and black arrow shows cytoplasmic staining. A and B are 20x, C and D are 40x magnification.

The Microcephalin and ASPM antibodies were initially optimized for use on paraffin-embedded TMA sections. Immunohistochemistry with these antibodies demonstrated that Microcephalin and ASPM were localized in the nucleus and in the cytoplasm of both the normal (Figure 1) and EOC tissue samples (Figure 2). The nuclear Microcephalin staining pattern was abolished when the antibody was pre-incubated in the presence of the peptide. However, some uniform low level background cytoplasmic staining was still present in all cores (Figure S1). Therefore, we have not included the cytoplasmic Microcephalin staining in any further analysis.

The staining pattern for ASPM antibody 279.3 performed in parallel to the staining with antibody 216.1 revealed that cytoplasmic staining was similar for both antibodies. However, 216.1 showed a more pronounced nuclear staining pattern than 279.3 and consequently this antibody was used to stain the EOC validation set. Results for staining patterns of representative cores and a summary of the staining results are shown in Figure S2 and Table S1.

To further confirm the validity of the antibodies to stain paraffin-embedded tissues, the antibodies were tested on paraffin-embedded U-2 OS cells with and without siRNA knockdown for Microcephalin or ASPM. Knockdown cells for both genes showed very weak staining relative to the control siRNA (Figure S3).

### Microcephalin Expression in Normal and EOC Tissue Samples

**Figure 3:**
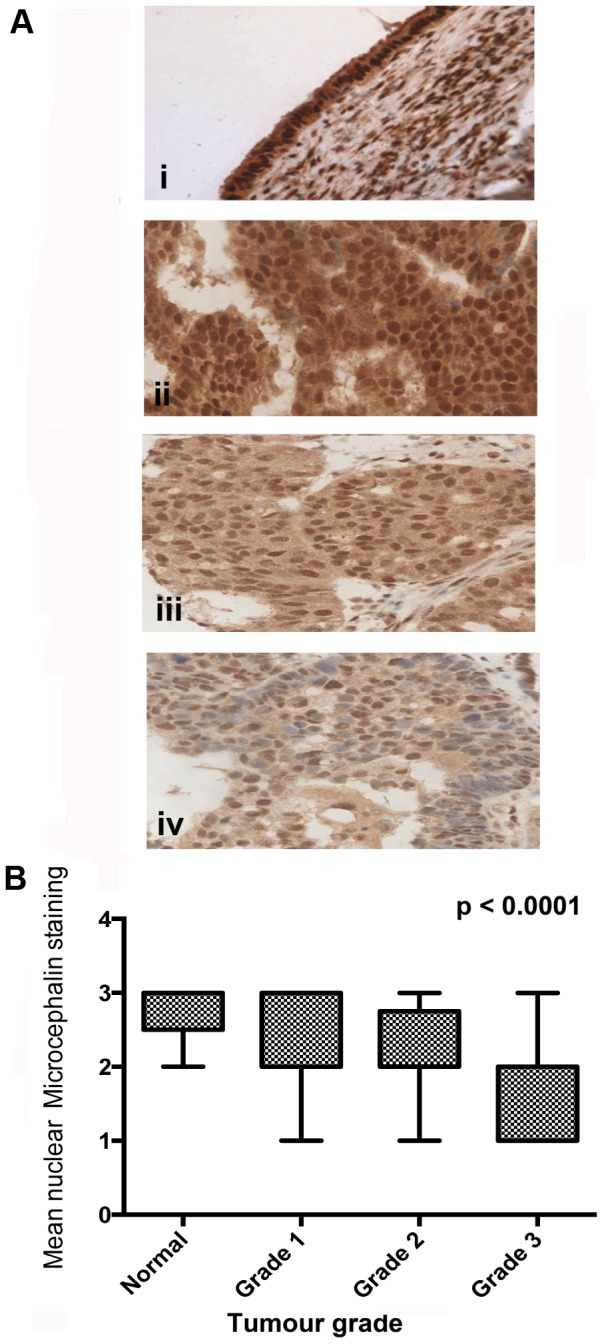
Microcephalin expression in EOC validation set samples correlates with tumour grade. Ai. Normal ovarian epithelial tissue with high Microcephalin expression. Aii-iv. Adenocarcinoma TMA cores showing strong Microcephalin expression in a low grade tumour (Aii), moderate Microcephalin in grade 2 (Aiii) and low levels of nuclear Microcephalin expression in a high grade tumour (Aiv). All images are 40x magnification. B. The correlation between nuclear Microcephalin expression decreases with increasing tumor grade (*p*<0.0001 using an ANOVA test).

**Figure 4:**
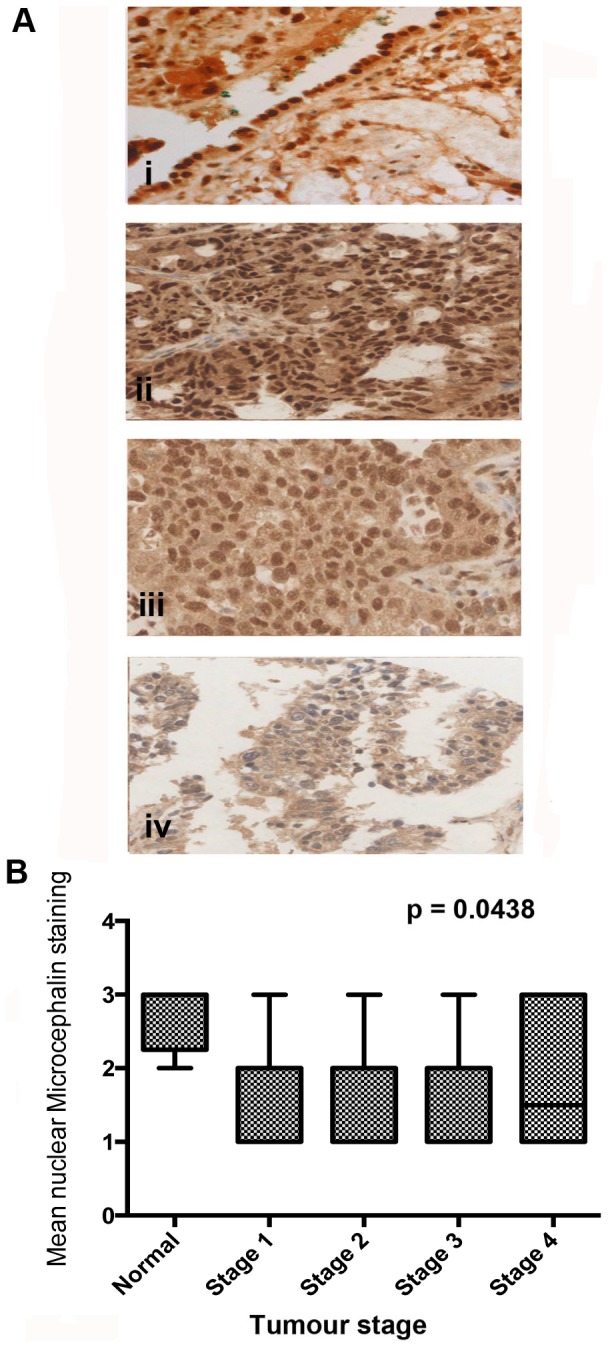
Microcephalin expression in EOC validation set samples inversely correlates with tumour stage. Ai. Normal ovarian epithelial tissue demonstrating high Microcephalin expression levels. Aii-4iv Adenocarcinoma TMA cores showing strong Microcephalin (Aii), moderate Microcephalin (Aiii) and low levels of nuclear Microcephalin expression (Aiv) in stage 1, 2 and 3 tumours respectively. All images are 40x magnification. B. Weak Microcephalin levels in the nucleus are associated with advanced tumour stage (*p* = 0.0438 using an ANOVA test).

**Figure 5:**
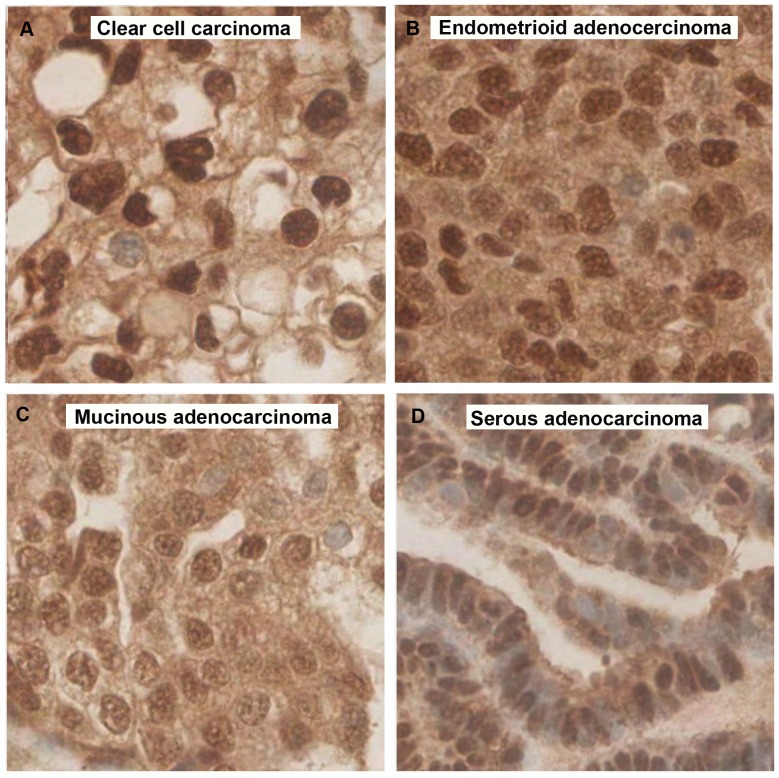
Microcephalin expression in different EOC subtypes. A. Clear cell carcinoma and B. Endometrioid adenocarcinoma both showing strong Microcephalin expression. C. Mucinous adenocarcinoma and D. Serous adenocarcinoma both presenting weak Microcephalin expression. All images are x40 magnification.

Normal ovarian, endometrium and fallopian tube tissue samples all revealed strong nuclear Microcephalin staining. The majority (90%) of normal ovarian epithelial cells demonstrated positive Microcephalin staining while this was slightly lower (70–80%) in the endometrium and fallopian tube samples (Figure 1A–F). Initially, the expression of Microcephalin was evaluated in the in house training set TMA. Dichotomous analysis in the training set demonstrated loss of Microcephalin expression in 16/22 (73%) cases (Table 2). In the EOC validation set, low Microcephalin expression was identified in 30% (89/294) of EOC samples (Table 3). Since we were particularly interested in Microcephalin expression levels and its association with tumourigenesis, we examined our IHC data to identify potential correlations with the associated clinical data. Interestingly, dichotomous analysis in the training set demonstrated loss of Microcephalin expression in grade 3 EOC tumours (Table 2). This finding was confirmed in the validation set with low nuclear staining mainly found in the grade 3 tumours (48%; 53/110 p<0.0001). In low-grade tumours, moderate or high nuclear staining was observed (*p*<0.0001) (Figure 3). Microcephalin nuclear expression also declined with increased tumour stage (*p* = 0.0438) (Figure 4). Patients over 50 years of age demonstrated lower nuclear Microcephalin levels (Table 3), but the difference was not statistically significant (*p* = 0.0581). No significant changes in Microcephalin expression were identified between different pathological subtypes (*p* = 0.486) (Table 3). However, the majority (8/10, 80%) of clear cell carcinomas displayed moderate or strong Microcephalin immunostaining. This may reflect the fact that 6/8 of these cases were stage I and or that the different subtypes of EOC follow different molecular pathways. We also noted high rates of loss of Microcephalin in serous and mucinous adenocarcinoma (68/207, 33% and 15/44, 34%, respectively, Table 3). Figure 5A–D demonstrates the expression of Microcephalin in the different epithelial ovarian cancer subtypes.

**Table 2:** Microcephalin nuclear intensity in the training set.

	Microcephalin nuclear intensity		
	N = 22	Low	Moderate
	(%)	(%)	(%)
**Tumour grade**			
G1	1 (4)	1 (100)	0 (0)
G2	5 (23)	4 (80)	1 (20)
G3	16 (73)	11 (69)	5 (31)
**Histological tumour type**			
Serous adenocarcinoma	11 (50)	8 (73)	3 (27)
Mucinous adenocarcinoma	2 (9)	2 (100)	0 (0)
Endometrioid adenocarcinoma	8 (36)	5 (62.5)	3 (37.5)
Carcinosarcoma	1 (4)	1 (100)	0 (0)

**Table 3:** Correlation of nuclear Microcephalin expression with clinicopathological data in the EOC validation set.

Malignant cases in the validation set	Microcephalin nuclear intensity					Statistical analysis
	Samples		Low	Moderate	High	P value
	N = 294 (%)	Valid n	(%)	(%)	(%)	
**Age distribution**						
<50	125 (42.5)	119	43 (36)	56 (47)	20 (17)	
≥50	169 (57.5)	159	46 (29)	70 (44)	43 (27)	0.0581
**Histological tumour type**						
Serous adenocarcinoma	209 (71.1)	208	69 (33)	90 (43)	49 (24)	
Mucinous adenocarcinoma	45 (15.3)	44	15 (34)	21 (45)	8 (18)	
Endometrioid adenocarcinoma	12 (4.1)	11	3 (27)	7 (46)	1 (9)	0.486
Adenocarcinoma	15 (5.1)	5	0 (0)	4 (80)	1 (20)	
Clear cell carcinoma	13 (4.4)	10	2 (20)	4 (40)	4 (40)	
**Tumour grade**						
G1	63 (25.7)	61	14 (23)	28 (46)	19 (31)	
G2	72 (29.3)	72	16 (22)	38 (53)	18 (25)	
G3	110 (45)	110	53 (48)	41 (37)	16 (15)	**<0.0001**
G2/G3	7	7	1	4	2	
Data Missing	42	28	5	15	8	
**Tumour FIGO stage**						
I	205 (69.7)	196	56 (29)	92 (47)	48 (24)	
II	42 (14.3)	38	12 (32)	19 (50)	7 (18)	**0.0438**
III	35 (11.9)	34	16 (47)	13 (38)	5 (15)	
IV	12 (4.1)	10	5 (50)	2 (20)	3 (30)	
**Tumour TNM stage**						
**Primary tumour invasion (T)**						
T1	208 (70.7)	199	57 (28.7)	93 (46.7)	49 (24.6)	T1 vs. T2 vs. T3 0.144
T2	59 (20.1)	54	21 (38.9)	25 (46.3)	8 (14.8)	Control vs. T1 **0.0021**
T3	27 (9.2)	25	11 (44)	8 (32)	6 (24)	Control vs. T2 **0.0009**
**Lymph node status**						
N0	257 (87)	242	74 (30)	112 (47)	56 (23)	
N1	36 (12.3)	35	15 (43)	14 (40)	6 (17)	0.1162
NX	1 (0.7)	1	0 (0)	0 (0)	1 (100)	
**Distant metastases**						
No (M0)	282 (95.9)	268	84 (31.5)	124 (46)	60 (22.5)	0.5251
Yes (M1)	12 (4.1)	10	5 (50)	2 (20)	3 (30)	

P≤0.05 is significant and are shown in bold.

### ASPM Expression in Normal and EOC Tissue Samples

The normal ovarian epithelium, endometrium and fallopian tube showed variable ASPM expression, with moderate to high nuclear expression and low to moderate cytoplasmic ASPM staining (Figure 1G–L). The ASPM expression pattern in EOC was determined by immunohistochemistry using the training set of 25 paraffin-embedded sections. The staining pattern in these tumour samples was nuclear and cytoplasmic. In the training set, high nuclear staining (combined medium and strong staining pattern) was observed in high-grade tumours (9/14, 64.3%), whereas high cytoplasmic ASPM was present in all cores regardless of grade. However, subdividing staining into weak, medium and strong revealed an increase in high nuclear ASPM levels in the grade 3 tumours. Overall, the proportion of positive nuclear staining was categorized into strong (21%, 4/19), medium (31.6%, 6/19) and weak (47.4%, 9/19) (Table 4). Analysis showed that 10.5% (2/19) of samples had strong cytoplasmic staining and 89.5% (17/19) had moderate cytoplasmic staining. Low cytoplasmic staining was not observed. Further analysis revealed a trend for increased cytoplasmic and nuclear ASPM staining with grade (Table 4).

**Table 4:** ASPM nuclear and cytoplasmic intensity in the training set.

	ASPM nuclear intensity				ASPM cytoplasmic intensity			
	N = 19	Low	Moderate	High	N = 19	Low	Moderate	High
	(%)	(%)	(%)	(%)	(%)	(%)	(%)	(%)
**Tumour grade**								
G1	2 (10.5)	2 (100)	0 (0)	0 (0)	2 (10.5)	0 (0)	2 (100)	0 (0)
G2	3 (15.8)	2 (66.7)	1 (33.3)	0 (0)	3 (15.8)	0 (0)	3 (100)	0 (0)
G3	14 (73.7)	5 (35.7)	5 (35.7)	4 (28.6)	14 (73.7)	0 (0)	12 (85.7)	2 (14.3)
**Histological tumour type**								
Serous adenocarcinoma	11 (58)	4 (36)	4 (36)	3 (27)	11 (58)	0 (0)	9 (82)	2 (18)
Mucinous adenocarcinoma	1 (5.3)	1 (100)	0 (0)	0 (0)	1 (5.3)	0 (0)	1 (100)	0 (0)
Endometrioid adenocarcinoma	6 (31.6)	3 (50)	2 (33.3)	1 (16.7)	6 (31.6)	0 (0)	6 (100)	0 (0)
Carcinosarcoma	1 (5.4)	1 (100)	0 (0)	0 (0)	1 (5.4)	0 (0)	1 (100)	0 (0)

**Figure 6:**
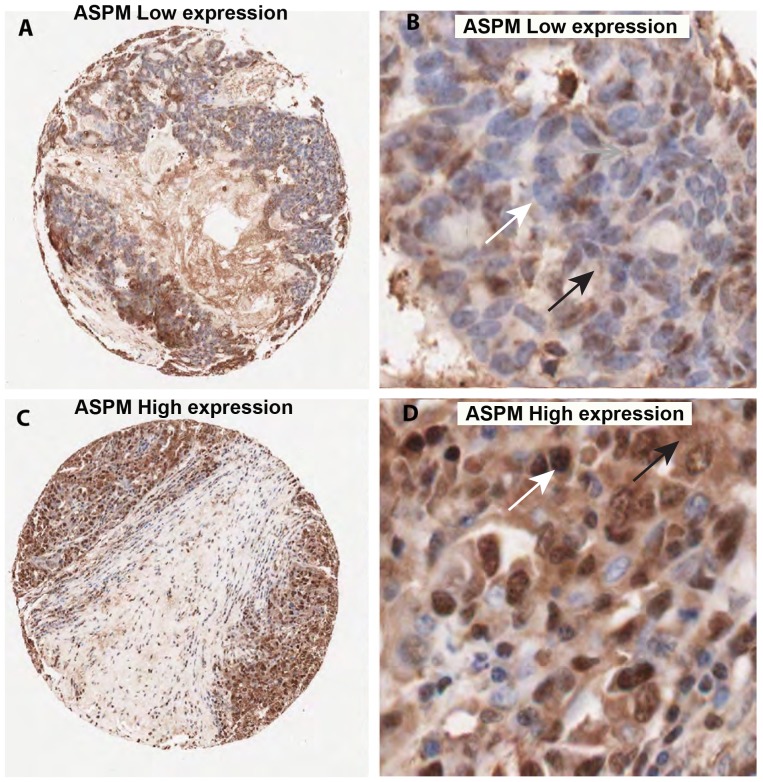
Variation of ASPM nuclear and cytoplasmic staining in validation set EOC tissue samples. A and B. TMA core with low nuclear and cytoplasmic ASPM expression. C and D. TMA core with high nuclear and cytoplasmic ASPM expression. White arrow indicates nuclear stain, black arrow indicates cytoplasmic stain. A and C are 6.2x and B, D are 20x magnification respectively.

**Figure 7:**
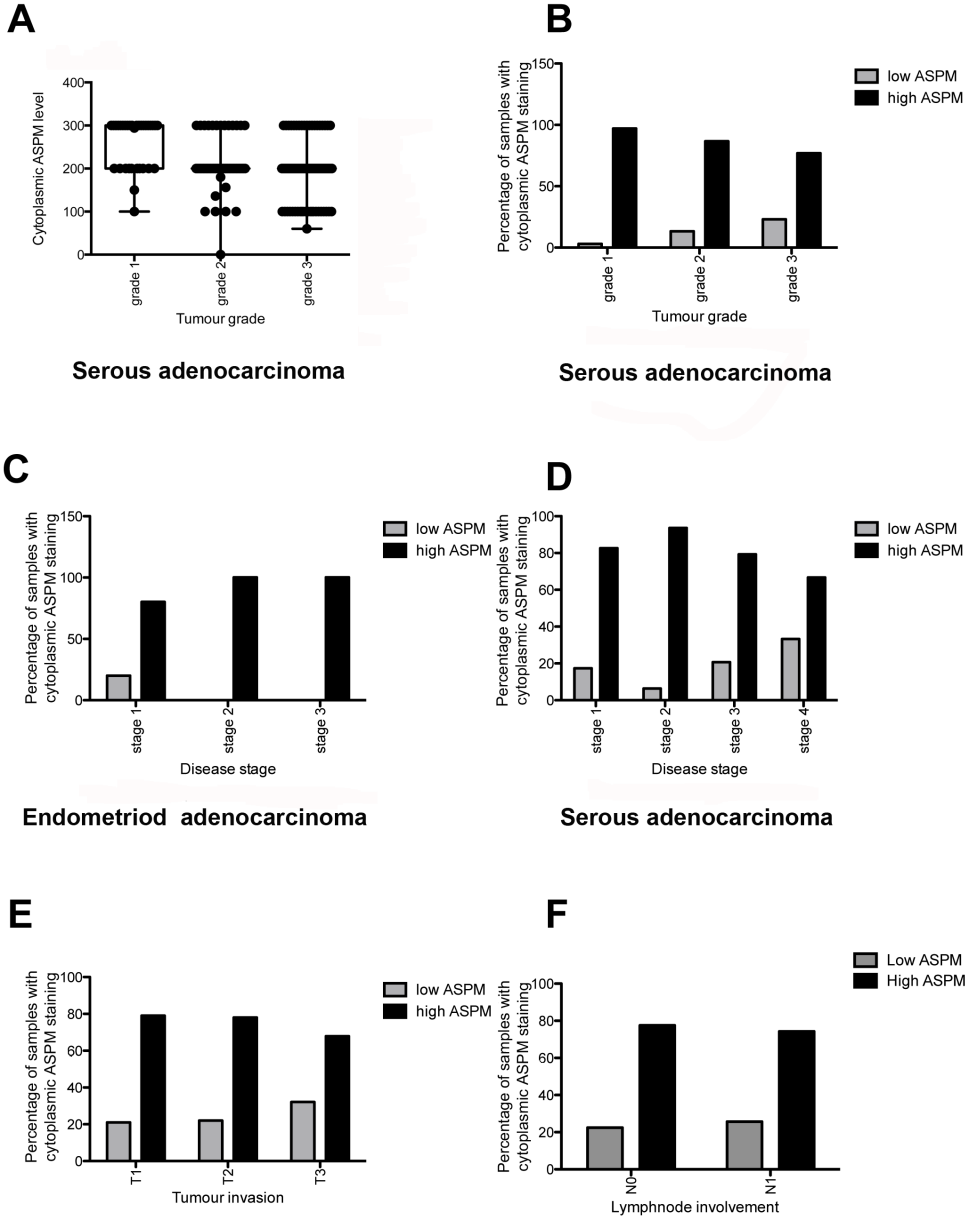
Cytoplasmic ASPM levels correlate with various clinic-pathological parameters in EOC. A. Cytoplasmic ASPM levels decrease with grade in serous EOC (continuous data, p<0.0001). B. Cytoplasmic ASPM levels decrease with tumour grade in the serous subtype (discontinuous data, p = 0.0239). C. Cytoplasmic ASPM levels increase with disease stage in endometrioid EOC (discontinuous data, p = 0.0229). D. Cytoplasmic ASPM levels decrease with disease stage in the serous subtype (discontinuous data, p = 0.0017). E. Cytoplasmic ASPM levels decrease with tumour invasion (discontinuous data, p = 0.02). F. High cytoplasmic ASPM levels correlate with no lymph node involvement (p = 0.04). All data analysed using an ANOVA test.

In the validation set nuclear and cytoplasmic staining was also observed. The staining patterns differed between tumour samples and examples of low and high ASPM expression are shown in Figure 6A–D. No statistically significant associations were identified between nuclear ASPM expression and the clinical data. Though, interestingly 52/294 (18%) of cases showed high nuclear ASPM expression, which increased with grade. Analysis of the validation set using continuous data analysis revealed low levels of cytoplasmic ASPM significantly correlated with high-grade tumours in the serous subtype (p<0.001) (Figure 7A). Discontinuous analysis revealed that there was a significant correlation between low cytoplasmic ASPM and high tumour grade (p = 0.0138) and also tumour FIGO stage (p = 0.032) (Table 5). Further analysis of the discontinuous data by subdivision into cancer subtypes revealed that in addition to the correlation of low cytoplasmic ASPM with high tumour grade, there was also an association of decreasing ASPM cytoplasmic levels with increasing grade in the serous subtype (p<0.0001) (Figure 7B) and an increase of ASPM expression levels with increasing tumour stage in the endometrioid subtype (p = 0.0229) (Figure 7C). A significant association between decreasing cytoplasmic ASPM staining and stage in the serous subtype was also identified (p = 0.0017) (Figure 7D). A significant association between cytoplasmic ASPM levels and patient age was not observed (Table 5).

**Table 5:** Correlation of cytoplasmic ASPM expression with clinicopathological data in the EOC validation set.

Malignant cases in the validation set	Statistical analysis				
	Samples		Low	High	P value
	N = 294 (%)	Valid n	(%)	(%)	
**Age distribution**					
<50	155 (52.7)	155	30 (19.3)	125 (80.7)	
≥50	139 (47.3)	139	36 (25.9)	103 (74.1)	0.0763
**Histological tumour type**					
Serous adenocarcinoma	209 (71.1)	198	33 (16.8)	165 (83.3)	
Mucinous adenocarcinoma	45 (15.3)	44	15 (34)	24 (57.2)	
Endometrioid adenocarcinoma	12 (4.1)	11	3 (27)	6 (54.6)	0.1643
Adenocarcinoma	15 (5.1)	5	0 (0)	7 (46.7)	
Clear cell carcinoma	13 (4.4)	10	2 (20)	4 (46.2)	
**Tumour grade**					
G1	61 (24.5)	61	8 (13.1)	53 (86.9)	
G2	78 (31.3)	78	18 (23.1)	60 (76.9)	
G3	110 (44.2)	110	26 (23.6)	84 (76.4)	**<0.0138**
G2/G3	7	5	0 (0)	5 (100)	
Data Missing	38	37	19 (51.4)	18 (48.7)	
**Tumour FIGO stage**					
I	205 (69.7)	196	40 (20.5)	155 (79.5)	
II	42 (14.3)	37	6 (16.2)	31 (83.7)	**0.0032**
III	35 (11.9)	34	9 (26.5)	25 (73.5)	
IV	12 (4.1)	10	3 (30)	7 (70)	
**Tumour TNM stage**					
**Primary tumour invasion (T)**					
T1	208 (70.7)	205	43 (21)	162 (79)	T1 vs. T2 vs. T3 **0.0198**
T2	59 (20.1)	59	13 (22)	46 (78)	
T3	27 (9.2)	27	8 (32.1)	19 (67.9)	
**Lymph node status**					
N0	257 (87)	254	57 (22.4)	197 (77.6)	
N1	36 (12.3)	35	9 (25.7)	26 (74.3)	**0.0401**
NX	1 (0.7)	1	0 (0)	1 (100)	
**Distant metastases**					
No (M0)	282 (95.9)	280	4 (33.3)	218 (77.2)	0.1568
Yes (M1)	12 (4.1)	12	62 (22.1)	8 (66.7)	

P≤0.05 is significant and are shown in bold.

### Relationship Between Histopathological Staging (FIGO and TNM staging) and Microcephalin and ASPM expression

In the validation set Microcephalin was found to decrease with increasing tumour stage when using the FIGO staging system (*p* = 0.0438) (Table 3). Similarly ASPM cytoplasmic staining decreased with increasing tumour stage when using the FIGO staging system (*p* = 0.0032) (Table 5). Microcephalin and ASPM expression levels were further assessed in the context of disease severity as determined by the level of tumour invasiveness (T1, T2 and T3). Nuclear Microcephalin expression levels decreased even if the tumour was limited to the ovaries (T1; p = 0.0021). When comparing Microcephalin expression in T2 with the control group, the result became more significant (p = 0.0009). However, there was no significant difference in Microcephalin expression when samples in the T1, T2 and T3 groups were compared to each other. Furthermore, Microcephalin expression did not significantly correlate with regional lymph node involvement (p = 0.1162) or distant metastasis (p = 0.5251) (Table 3).

For ASPM, an association between cytoplasmic staining levels and tumour invasiveness was identified. High cytoplasmic ASPM levels were found at T1, which decreased significantly with T2 and T3 (p = 0.0198) (Figure 7E). High cytoplasmic ASPM also correlated with no lymph node involvement (N0) and it decreased with lymph node involvement (N1) (p = 0.04) (Figure 7F) (Table 5).

## Discussion

EOC is frequently first diagnosed at an advanced stage due to the lack of symptoms and reliable early detection methods. Consequently the identification of diagnostic and prognostic biomarkers for EOC is of major clinical importance. In this study we investigated the expression of two MCPH proteins, Microcephalin and ASPM, in EOC tumour tissue samples. EOC displays a high degree of aneuploidy. DNA damage response proteins are a vital defence against genomic instability and defects in these proteins may lead to cancer. Microcephalin has a known role in DNA repair and ASPM for spindle function during mitosis. The purpose of this study was to determine whether Microcephalin and ASPM expression are associated with clinicopathological parameters in patients with EOC. Both proteins had been shown to be deregulated in other cancers in a manner that was associated with tumour progression [16], [28]–[30], [36].

Recently we reported an association of Microcephalin and ASPM levels in malignant cells derived from ascitic fluids from EOC patients with various clinic-pathological parameters [36]. In the present, larger scale study, nuclear and/or cytoplasmic Microcephalin staining was identified in the tumour cells. Our results in the training set revealed a reduction in nuclear Microcephalin expression in 73% (16/22) of EOC tumours; this percentage was reduced to 30% (89/294) in the validation set. This difference between the two cohorts potentially reflects the higher number of grade 3 cases in the training set (73%; 16/22) compared to the validation set (37%; 110/294). In this study low Microcephalin expression was identified in high grade and advanced stage tumours (*p*<0.0001 and p = 0.0438 respectively). These findings match our previous study on ovarian ascites samples that indicated that Microcephalin expression was reduced in cell cultures derived from ascites of EOC patients with advanced tumours [36]. Our results are compatible with studies that reporting reduced *MCPH1* DNA copy number in 72% (39/54) of breast cancers [16] and to our own findings of reduced Microcephalin expression in 93/319 (29%) of breast cancer samples, particularly in the higher grade tumours [28].

Previously, we identified a correlation between the abnormal localization of Microcephalin with tumour grade in primary cultures of malignant cells derived from ascitic fluids from patients with EOC. In these cells, cytoplasmic Microcephalin increased with tumour grade. We suggested that might be because of *MCPH1* deletion mutations in the C-terminal BRCT domains that have previously been shown to result in Microcephalin moving from a nuclear to cytoplasmic localization similar to *BRCA1* [41], [42], [43]. A recent study characterizing different *MCPH1* splice variants reported mutation or deletion of nuclear localization signals within the *MCPH1* gene resulted in a localization change from nuclear to cytoplasmic [43]. In this study weak to moderate Microcephalin cytoplasmic staining was seen in all grade 2 and 3 samples and increased cytoplasmic Microcephalin expression was associated with increased tumour grade (*p* = 0.0051). However due to non-specific background cytoplasmic staining observed in the blocking peptide experiment, cytoplasmic expression was not included here.

Our findings of reduced expression of the DNA repair protein Microcephalin in high grade tumours but few low grade tumours is consistent with the molecular characteristics of these different tumour types. Loss of DNA repair function e.g. BRCA genes leading to chromosomal instability and a complex genome is common in high grade cancers. In contrast to low grade tumours which do not tend to display chromosomal instability and BRCA mutations.

Similarly to Microcephalin expression, in this study we recapitulated our recent findings on cytoplasmic ASPM levels and tumour grade. In primary cultures of malignant cells derived from ovarian ascites samples we had determined that cytoplasmic ASPM levels decreased with tumour grade. In this study we were able to verify this result in the serous subtype in the large-scale validation set of EOC tumour samples. In addition we were able to determine associations of cytoplasmic ASPM levels with disease stage, where high cytoplasmic ASPM levels were predominantly found in low stages (1 and 2) in the serous subtype and an increase of cytoplasmic ASPM levels with stage in the endometrioid subtype. It has been suggested that the different subtypes of EOC follow different molecular pathways and this may be represented in our results. Cytoplasmic ASPM levels were also found to be associated with tumour invasiveness with highest cytoplasmic ASPM levels found only when the tumour remained confined to the ovaries (T1). This may indicate that high levels of cytoplasmic ASPM are important for tumourigenesis but not for tumour progression.

Although cytoplasmic and nuclear locations of ASPM in other tumour types have been noted in the literature [32], [44] so far there is little information about the role of this differential location of ASPM, especially with regards to EOC. It has been demonstrated that there are at least four isoforms of ASPM with potentially different functions [24]. Our ASPM antibody should detect expression of full length and ASPM variant 1 [24], however little research has been performed on the relative cellular distribution or relative expression levels of the ASPM isoforms. In EOC tumours, ASPM over expression and/or the increase in ASPM cytoplasmic localisation may be explained as an increase in relative expression of only one of the two detectable ASPM isoforms, or of a novel isoform and the change in expression of the fully functional wtASPM protein in relation to the partially functional ASPM isoform may have an affect on cancer progression.

Interestingly, our findings of different cytoplasmic ASPM expression levels and associations with tumour stage among two subtypes reflect recent findings in the literature. For example, Köbel et al, 2008 [45], stated that a study of 21 candidate biomarkers for EOC revealed a varied association of biomarker expression with subtypes and they argued that each subtype within a cohort should be analyzed discretely. In our case the associations of ASPM expression levels especially in the serous and endometrioid subtypes may hint at distinct roles of ASPM in these cancer types. In the case of serous EOC ASPM may play a role especially in the tumorigenesis of high grade serous carcinomas which are characterised by high chromosomal instabilty and aneuploidy. In endometrioid cancers this role may be different as most of our samples were low grade and there seemed to be an increase in cytoplasmic ASPM with stage. It will be interesting to confirm our findings in a larger data set of the less represented EOC subtypes. Our finding support the notion that EOC is not a single disease entity but should be managed as several distinct disease entities.

In summary, in this study we investigated the potential use of Microcephalin and ASPM expression in clinical practice in EOC in a large scale study. We conclude that deregulation of Microcephalin and ASPM expression is significantly associated with tumourigenesis. The results from this study warrant the further investigation of Microcephalin and ASPM as potential biomarkers in EOC.

## Supporting Information

Figure S1 **Characterization of the anti-Microcephalin antibody.** Immunohistochemical staining of Microcephalin in the absence (A and C) and presence (B and D) of the peptide the antibody was raised against illustrating, low non-specific cytoplasmic background staining. A and B are 40x magnification, C and D are 10x magnification.
(TIF)

Figure S2 **Comparison of two different ASPM antibodies.** The staining patterns of the *N*-terminal 216.1 and *C*-terminal 279.3 ASPM antibodies 216.1 (A and C) and 279.3 (B and D) revealed identical cytoplasmic and nuclear localization and relative protein expression levels. A and B TMA core obtained for the same patient sample showing low level ASPM expression. C and D TMA core obtained for the same patient showing high ASPM expression. All images are 6.2x magnification.
(TIF)

Figure S3 **Conformation of antibody specificity by siRNA knockdown in U-2 0S cells.** Immunohistochemistry analysis of paraffin embedded U-2 0S cells after siRNA knockdown stained with anti Microcephalin (A and B) or ASPM (C and D) antibodies respectively. Cells transfected with scrambled control siRNA showed high Microcephalin (A) and ASPM (C) expression respectively. Cells transfected with *MCPH1* (B) or *ASPM* (D) siRNA showed low expression. All images are x40 magnification.
(TIF)

Table S1 **Analysis of the staining intensities of two different ASPM antibodies 216.1 and 279.3 performed on the training set TMA.**
(DOCX)
