# Supplementary figures and images for: Deregulation of Microcephalin and ASPM Expression Are Correlated with Epithelial Ovarian Cancer Progression
Source: PLoS One. 2014 May 15;9(5):e97059. doi: 10.1371/journal.pone.0097059 (PMC4022499; doi:10.1371/journal.pone.0097059)

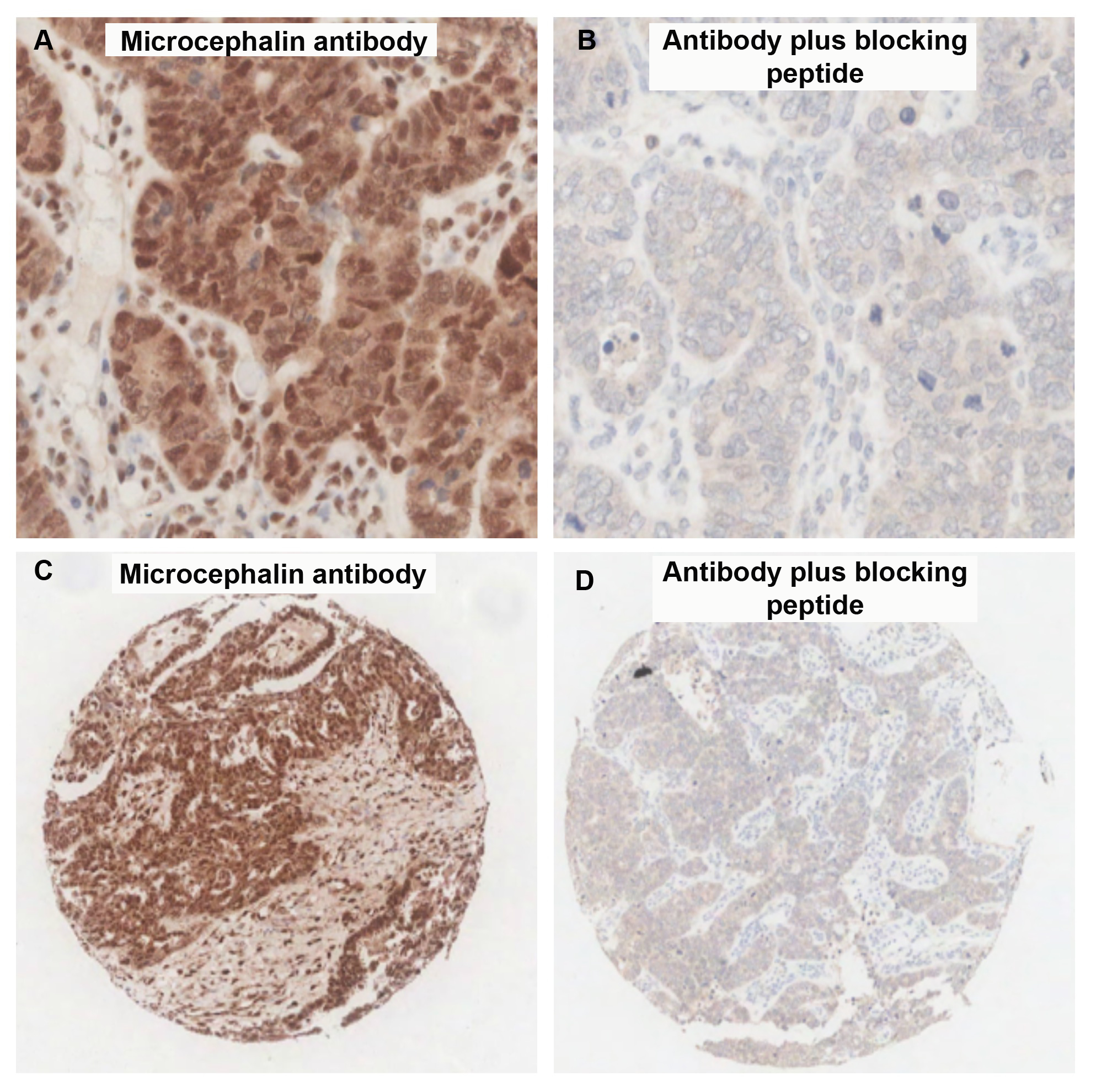

Supplement: Figure S1 — Characterization of the anti-Microcephalin antibody. Immunohistochemical staining of Microcephalin in the absence (A and C) and presence (B and D) of the peptide the antibody was raised against illustrating, low non-specific cytoplasmic background staining. A and B are 40x magnification, C and D are 10x magnification. (TIF) [file pone.0097059.s001.tif]

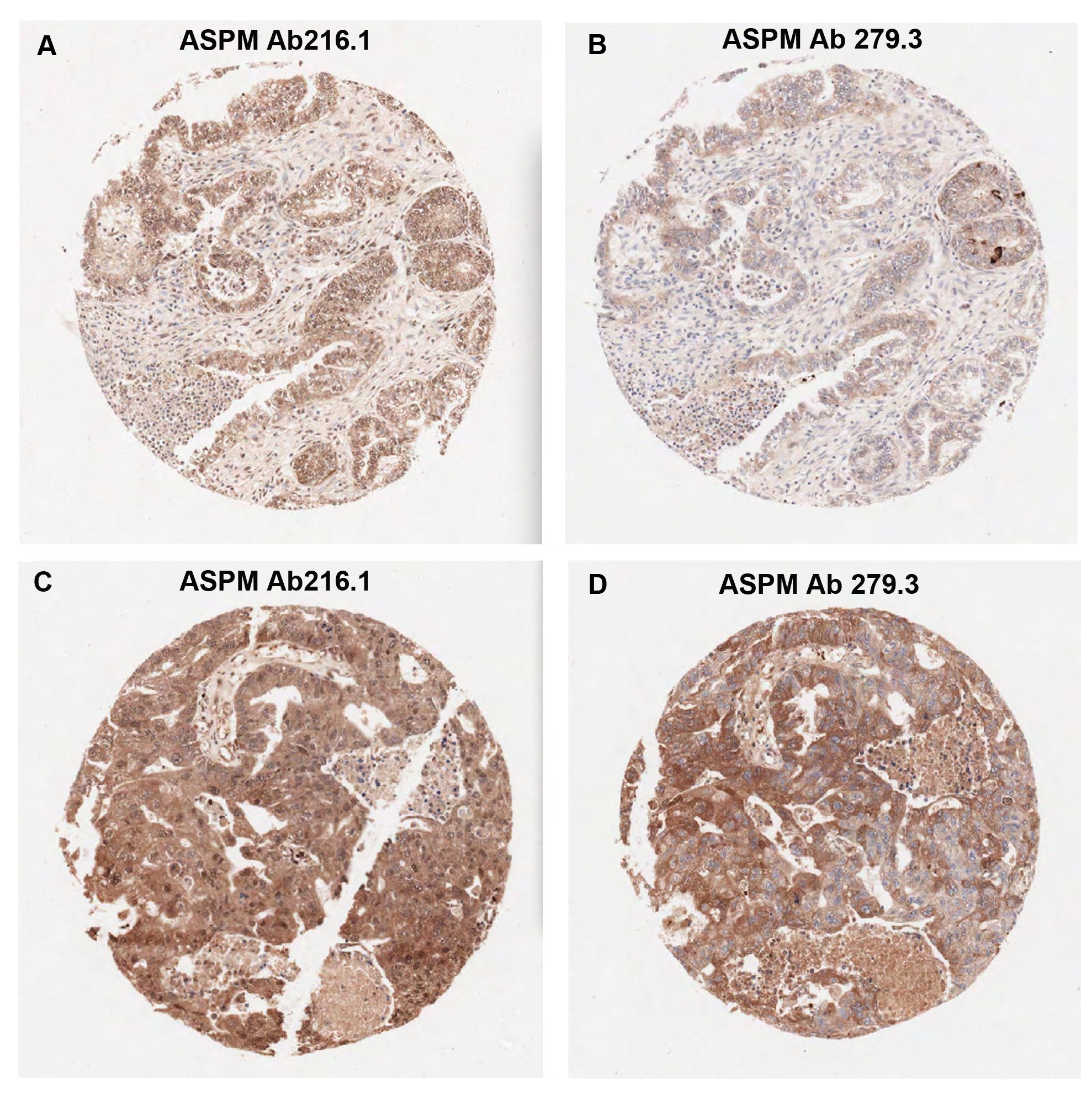

Supplement: Figure S2 — Comparison of two different ASPM antibodies. The staining patterns of the N-terminal 216.1 and C-terminal 279.3 ASPM antibodies 216.1 (A and C) and 279.3 (B and D) revealed identical cytoplasmic and nuclear localization and relative protein expression levels. A and B TMA core obtained for the same patient sample showing low level ASPM expression. C and D TMA core obtained for the same patient showing high ASPM expression. All images are 6.2x magnification. (TIF) [file pone.0097059.s002.tif]

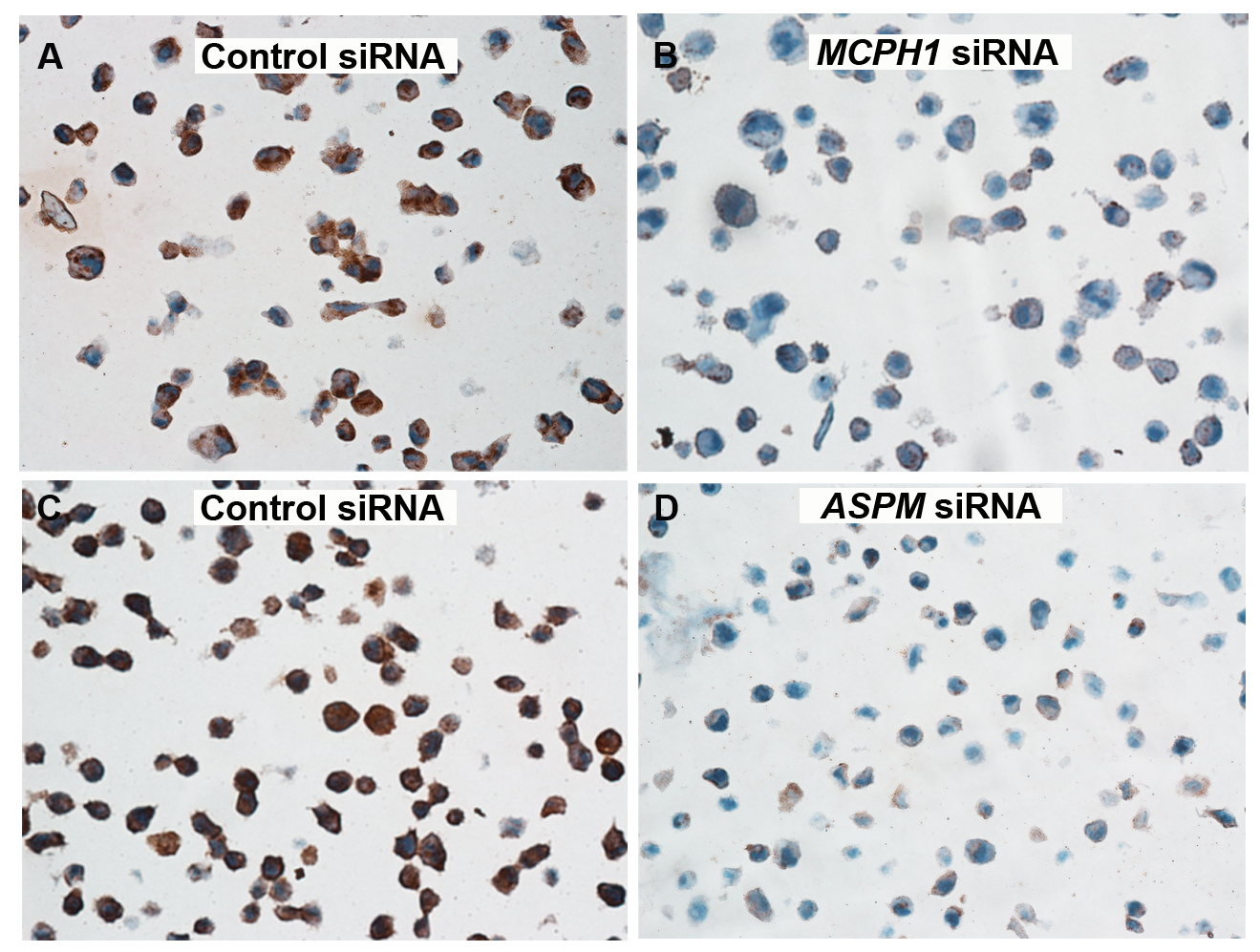

Supplement: Figure S3 — Conformation of antibody specificity by siRNA knockdown in U-2 0S cells. Immunohistochemistry analysis of paraffin embedded U-2 0S cells after siRNA knockdown stained with anti Microcephalin (A and B) or ASPM (C and D) antibodies respectively. Cells transfected with scrambled control siRNA showed high Microcephalin (A) and ASPM (C) expression respectively. Cells transfected with MCPH1 (B) or ASPM (D) siRNA showed low expression. All images are x40 magnification. (TIF) [file pone.0097059.s003.tif]
