# Supplementary material for: Deregulation of Microcephalin and ASPM Expression Are Correlated with Epithelial Ovarian Cancer Progression
Source: PLoS One. 2014 May 15;9(5):e97059. doi: 10.1371/journal.pone.0097059 (PMC4022499; doi:10.1371/journal.pone.0097059)
Supplement: Table S1 — Analysis of the staining intensities of two different ASPM antibodies 216.1 and 279.3 performed on the training set TMA. (DOCX) [file pone.0097059.s004.docx]

**Supplementary Table S1.** **Analysis of the staining intensities of two different ASPM antibodies 216.1 and 279.3 performed on the training set TMA.**

| Sample ID | Antibody 279.3 | Antibody 216.1 |
| --- | --- | --- |
| 20536 | 2 | 2 |
| 15727 | No core | No core |
| 15874 | 2 | 2 |
| 3999 | 2 | 2 |
| 83083 | 2 | 2 |
| 3329 | 2 | 2 |
| 2324 | No core | No core |
| 7636 | 2 | 2 |
| 10275 | 1 | 2 |
| 7452 | 3 | 2 |
| 3467 | No cancer |  |
| 13679 | 3 | 2 |
| 10886 | 3 | 3 |
| 10131 | 2 | 2 |
| 16004 | 1 core, 3 | 1 core, 2 |
| 13453 | 2 | 2 |
| 9456 | No core | No core |
| 8371 | 2 | 2 |
| 11385 | 2 | 2 |
| 16943 | 3 | 3 |
| 18622 | 2 | 3 |
| 13961 | 2 | 3 |
| 19672 | 3 | 2 |
| 17957 | 2 | 2 |
| 9719 | No core | No core |
